# (2-Hydroxy­phenyl­imido-κ*N*)(methano­lato-κ*O*)[2-(2-oxidobenzyl­ideneamino)phenolato-κ^2^ *O*,*N*,*O*′](triphenyl­phosphine-κ*P*)rhenium(V)

**DOI:** 10.1107/S1600536808010684

**Published:** 2008-05-03

**Authors:** Jason P. Holland, Peter J. Barnard, Jonathan R. Dilworth, David J. Watkin

**Affiliations:** a Chemistry Research Laboratory, University of Oxford, 12 Mansfield Road, Oxford OX1 3TA, England; b Chemical Crystallography, Chemistry Research Laboratory, University of Oxford, 12 Mansfield Road, Oxford OX1 3TA, England

## Abstract

In the neutral title compound, [Re(C_6_H_5_NO)(C_13_H_9_NO_2_)(CH_3_O)(C_18_H_15_P)], an 18-valence-electron complex, the Re^V^ ion lies in an octa­hedral coordination geometry with the tridentate dianionic Schiff base 2-(2-oxidobenzyl­idene­amino)phenolate ligand occupying three equatorial coordination sites, and with the triphenyl­phosphine ligand situated *trans* to the imine N atom. The Re^V^ coordination is completed with a methano­late ligand and a 2-hydroxy­phenyl­imido(2-) ligand. There are two molecules in the asymmetric unit. The crystal structure involves O—H⋯O and C—H⋯O hydrogen bonds. One N and one C atom are disordered over two positions; the site occupancy factors are *ca* 0.7 and 0.3.

## Related literature

For related literature, see: Chen *et al.* (2000 ▶, 2001 ▶); Femia *et al.* (2001 ▶); Sheldrick (2008 ▶).
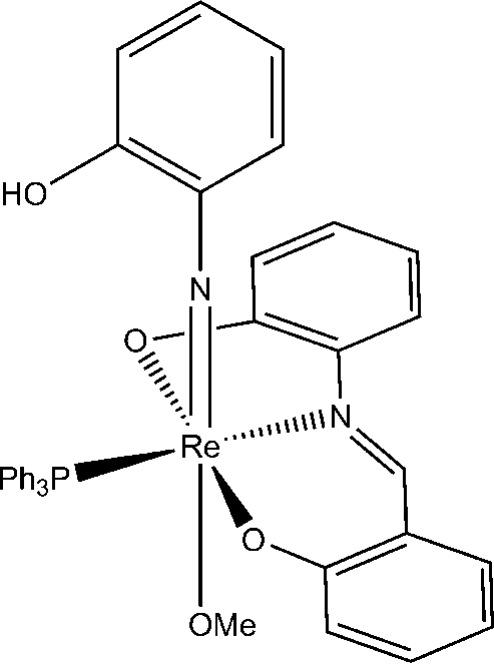


## Experimental

### 

#### Crystal data

[Re(C_6_H_5_NO)(C_13_H_9_NO_2_)(CH_3_O)(C_18_H_15_P)]
*M* *_r_* = 797.86
Monoclinic, 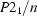

*a* = 10.0193 (2) Å
*b* = 32.9759 (3) Å
*c* = 19.4605 (2) Å
β = 90.1597 (4)°
*V* = 6429.64 (16) Å^3^
*Z* = 8
Mo *K*α radiation
μ = 3.87 mm^−1^
*T* = 150 K
0.24 × 0.24 × 0.06 mm

#### Data collection

Nonius KappaCCD diffractometer
Absorption correction: multi-scan (*DENZO*/*SCALEPACK*; Otwinowski & Minor, 1997 ▶) *T*_min_ = 0.39, *T*_max_ = 0.79
56004 measured reflections
14195 independent reflections
10958 reflections with *I* > 2σ(*I*)
*R*_int_ = 0.052

#### Refinement

*R*[*F* ^2^ > 2σ(*F* ^2^)] = 0.046
*wR*(*F* ^2^) = 0.085
*S* = 0.99
13952 reflections
847 parameters
614 restraints
H-atom parameters constrained
Δρ_max_ = 2.85 e Å^−3^
Δρ_min_ = −3.36 e Å^−3^

### 

Data collection: *COLLECT* (Nonius, 2001 ▶); cell refinement: *DENZO*/*SCALEPACK* (Otwinowski & Minor, 1997 ▶); data reduction: *DENZO*/*SCALEPACK*; program(s) used to solve structure: *SIR92* (Altomare *et al.*, 1994 ▶); program(s) used to refine structure: *CRYSTALS* (Betteridge *et al.*, 2003 ▶); molecular graphics: *CAMERON* (Watkin *et al.*, 1996 ▶); software used to prepare material for publication: *CRYSTALS*.

## Supplementary Material

Crystal structure: contains datablocks I, global. DOI: 10.1107/S1600536808010684/br2071sup1.cif

Structure factors: contains datablocks I. DOI: 10.1107/S1600536808010684/br2071Isup2.hkl

Additional supplementary materials:  crystallographic information; 3D view; checkCIF report

## Figures and Tables

**Table 1:** Hydrogen-bond geometry (Å, °)

*D*—H⋯*A*	*D*—H	H⋯*A*	*D*⋯*A*	*D*—H⋯*A*
O3—H31⋯O1	0.81	1.93	2.693 (8)	156
O7—H71⋯O5	0.83	1.89	2.720 (8)	177
C34—H341⋯O1	0.94	2.53	3.397 (8)	153
C60—H601⋯N4	0.94	2.59	3.342 (8)	137
C72—H721⋯O5	0.92	2.38	2.983 (8)	123

**Fig. 1.**
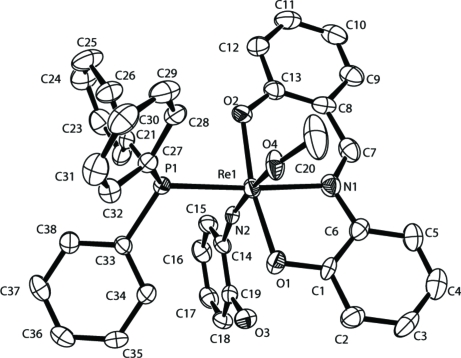
ORTEP representation of the title complex with displacement ellipsoids at 50% probability. H atoms have been omitted for clarity.

**Fig. 2.**
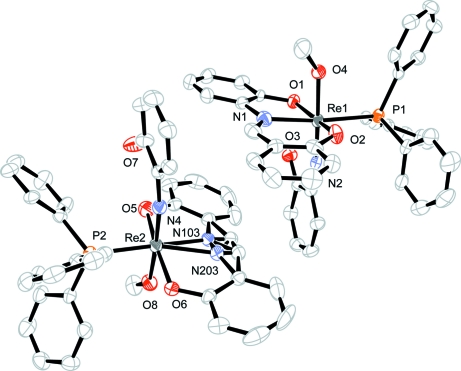
ORTEP representation (50% probability) of the two crystallographically distinct molecules in the asymmetric unit.

## supplementary crystallographic information

### Comment

The asymmetric unit contains two crystallographically distinct molecules, neither of which possess any crystallographic symmetry. These differ in the conformation of the PPh3 ligands. One of the molecules (Re2 *etc*.) exhibited unusually large thermal parameters for some of the atoms of the imine ligand, suggesting that it may be disordered. This was modelled satisfactorily by 'splitting' the nitrogen and one carbon atom. The hydroxyl groups of the coordinated 2-hydroxyphenylimido(2-) ligands form intramolecular hydrogen bonds to an oxygen atom of the imido ligand (O3···O1, 2.694 (6) Å and O7···O5, 2.720 (6) Å).

### Experimental

Dark-brown crystals of the neutral Re^V^ complex were isolated as a by-product from the crude reaction mixture after reacting [Re^III^Cl_3_(CH_3_CN)(PPh_3_)_2_] (0.40 g, 5.0 *x* 10 ^-4^ mol) with 3 equiv. of the Schiff base salicylaldehyde-2-hydroxyaniline (0.32 g, 1.5 *x* 10 ^-3^ mol) in the presence of excess triethylamine, in methanol (30 ml). After stirring under reflux for 8 h, the reaction mixture was allowed to cool for 12 h during which time crystals formed.

### Refinement

The structure contains two independent molecules in the asymmetric unit (*Z*'=2). On of them is well resolved except for a marginal Hirshfeld test on atoms C7—C8. In the other molecule, the [salicylaldehyde-2-hydroxyanilato(2-)] moiety is disordered with the atoms N103 and N203, and C145 and C245 being clearly distinguished in a difference map. Refinement of the disorder was achieved by the use of Marquardt shift limiting restraints (damping) and molecular geometry and thermal simlarity restraints.

The H atoms were all located in a difference map, but those attached to carbon atoms were repositioned geometrically. The H atoms were initially refined with soft restraints on the bond lengths and angles to regularize their geometry (C—H in the range 0.93–0.98, Å) and *U*_iso_(H) (in the range 1.2–1.5 times *U*_eq_ of the parent atom), after which the positions were refined with riding constraints.

### Crystal data

**Table:**

[Re(C_6_H_5_NO)(C_13_H_9_NO_2_)(CH_3_O)(C_18_H_15_P)]	*F*_000_ = 3168
*M**_r_* = 797.86	*D*_x_ = 1.648 Mg m^−^^3^
Monoclinic, *P*2_1_/*n*	Mo *K*α radiation λ = 0.71073 Å
Hall symbol: -P 2yn	Cell parameters from 56004 reflections
*a* = 10.0193 (2) Å	θ = 5–28º
*b* = 32.9759 (3) Å	µ = 3.87 mm^−^^1^
*c* = 19.4605 (2) Å	*T* = 150 K
β = 90.1597 (4)º	Plate, dark brown
*V* = 6429.64 (16) Å^3^	0.24 × 0.24 × 0.06 mm
*Z* = 8	

### Data collection

**Table:**

Area diffractometer	10958 reflections with *I* > 2σ(*I*)
Monochromator: graphite	*R*_int_ = 0.052
*T* = 150 K	θ_max_ = 27.6º
ω scans	θ_min_ = 5.2º
Absorption correction: multi-scan(DENZO/SCALEPACK; Otwinowski & Minor, 1997)	*h* = −12→12
*T*_min_ = 0.39, *T*_max_ = 0.79	*k* = −42→42
56004 measured reflections	*l* = −25→25
14195 independent reflections	

### Refinement

**Table:**

Refinement on *F*^2^	Primary atom site location: structure-invariant direct methods
Least-squares matrix: full	Hydrogen site location: inferred from neighbouring sites
*R*[*F*^2^ > 2σ(*F*^2^)] = 0.046	H-atom parameters constrained
*wR*(*F*^2^) = 0.085	Method = Modified Sheldrick (2008) *w* = 1/[σ^2^(*F*^2^) + (0.01*P*)^2^ + 33.25*P*], where *P* = [max(*F*_o_^2^,0) + 2*F*_c_^2^]/3
*S* = 0.99	(Δ/σ)_max_ = 0.002
13952 reflections	Δρ_max_ = 2.85 e Å^−^^3^
847 parameters	Δρ_min_ = −3.36 e Å^−^^3^
614 restraints	Extinction correction: None

### Fractional atomic coordinates and isotropic or equivalent isotropic displacement parameters (Å^2^)

**Table:**

	*x*	*y*	*z*	*U*_iso_*/*U*_eq_	Occ. (<1)
Re1	0.335488 (19)	0.426935 (7)	0.267904 (10)	0.0258	
O1	0.4009 (3)	0.45761 (11)	0.35638 (18)	0.0311	
C1	0.5212 (5)	0.44409 (16)	0.3784 (2)	0.0256	
C2	0.5808 (5)	0.46241 (18)	0.4355 (3)	0.0354	
C3	0.7041 (6)	0.4494 (2)	0.4585 (3)	0.0428	
C4	0.7718 (6)	0.4188 (2)	0.4258 (3)	0.0450	
C5	0.7142 (5)	0.40022 (18)	0.3692 (3)	0.0390	
C6	0.5893 (5)	0.41223 (17)	0.3462 (3)	0.0295	
N1	0.5143 (4)	0.39498 (14)	0.2899 (2)	0.0353	
C7	0.5504 (6)	0.36392 (18)	0.2578 (3)	0.0394	
C8	0.4841 (5)	0.34664 (17)	0.1976 (3)	0.0321	
C9	0.5415 (6)	0.31020 (18)	0.1743 (3)	0.0401	
C10	0.4927 (6)	0.2899 (2)	0.1185 (3)	0.0464	
C11	0.3827 (7)	0.3060 (2)	0.0845 (3)	0.0476	
C12	0.3247 (6)	0.34162 (19)	0.1052 (3)	0.0378	
C13	0.3724 (5)	0.36310 (17)	0.1623 (3)	0.0332	
O2	0.3101 (4)	0.39741 (12)	0.17743 (17)	0.0350	
N2	0.2287 (4)	0.39752 (13)	0.3194 (2)	0.0242	
C14	0.1583 (5)	0.37914 (16)	0.3718 (2)	0.0251	
C15	0.0806 (5)	0.34454 (16)	0.3589 (3)	0.0311	
C16	0.0163 (5)	0.32490 (18)	0.4116 (3)	0.0378	
C17	0.0291 (5)	0.33928 (18)	0.4783 (3)	0.0363	
C18	0.1056 (5)	0.37316 (17)	0.4923 (3)	0.0324	
C19	0.1705 (5)	0.39350 (16)	0.4391 (2)	0.0255	
O3	0.2464 (3)	0.42580 (11)	0.45571 (17)	0.0316	
O4	0.4528 (3)	0.46395 (12)	0.2215 (2)	0.0390	
C20	0.5704 (6)	0.4654 (3)	0.1904 (4)	0.0755	
P1	0.14834 (12)	0.46856 (4)	0.23254 (6)	0.0235	
C21	0.0321 (5)	0.43868 (16)	0.1788 (3)	0.0265	
C22	−0.0394 (5)	0.40837 (19)	0.2099 (3)	0.0389	
C23	−0.1214 (6)	0.3834 (2)	0.1718 (3)	0.0459	
C24	−0.1324 (7)	0.3893 (2)	0.1015 (4)	0.0552	
C25	−0.0620 (7)	0.4197 (2)	0.0704 (3)	0.0566	
C26	0.0197 (6)	0.44445 (19)	0.1090 (3)	0.0393	
C27	0.1911 (5)	0.51265 (16)	0.1801 (2)	0.0252	
C28	0.2798 (5)	0.50819 (18)	0.1253 (2)	0.0313	
C29	0.3110 (6)	0.54079 (19)	0.0842 (3)	0.0392	
C30	0.2544 (6)	0.57833 (19)	0.0968 (3)	0.0418	
C31	0.1678 (6)	0.58301 (18)	0.1505 (3)	0.0405	
C32	0.1367 (5)	0.55041 (17)	0.1929 (3)	0.0331	
C33	0.0393 (5)	0.48936 (15)	0.2985 (2)	0.0239	
C34	0.0928 (5)	0.50044 (16)	0.3615 (2)	0.0274	
C35	0.0119 (5)	0.51914 (16)	0.4101 (3)	0.0290	
C36	−0.1211 (5)	0.52736 (17)	0.3962 (3)	0.0352	
C37	−0.1735 (5)	0.51677 (18)	0.3336 (3)	0.0389	
C38	−0.0951 (5)	0.49755 (18)	0.2851 (3)	0.0343	
Re2	0.65712 (2)	0.188938 (7)	0.281907 (11)	0.0288	
O5	0.7172 (3)	0.23421 (11)	0.35005 (19)	0.0353	
C39	0.6234 (5)	0.26209 (16)	0.3650 (3)	0.0270	
C40	0.6492 (6)	0.29110 (18)	0.4152 (3)	0.0360	
C41	0.5526 (7)	0.31904 (18)	0.4336 (3)	0.0496	
C42	0.4290 (7)	0.3181 (2)	0.4027 (3)	0.0544	
C43	0.4026 (6)	0.2901 (2)	0.3520 (3)	0.0497	
C44	0.4983 (5)	0.26254 (16)	0.3323 (2)	0.0301	
N103	0.4943 (5)	0.22937 (17)	0.2813 (3)	0.0279	0.7292
C145	0.3975 (7)	0.2261 (2)	0.2390 (3)	0.0316	0.7292
C46	0.3818 (5)	0.19131 (16)	0.1954 (3)	0.0307	
C47	0.2645 (6)	0.1960 (2)	0.1550 (3)	0.0537	
C48	0.2186 (7)	0.1650 (3)	0.1142 (3)	0.0669	
C49	0.2873 (7)	0.1293 (3)	0.1119 (3)	0.0598	
C50	0.3991 (6)	0.12388 (19)	0.1494 (3)	0.0389	
C51	0.4511 (5)	0.15396 (17)	0.1918 (3)	0.0300	
O6	0.5628 (3)	0.14568 (11)	0.22653 (19)	0.0342	
N4	0.7487 (4)	0.21231 (13)	0.2162 (2)	0.0290	
C52	0.8353 (5)	0.23423 (16)	0.1770 (3)	0.0268	
C53	0.8459 (5)	0.22802 (17)	0.1053 (3)	0.0338	
C54	0.9336 (6)	0.25030 (18)	0.0662 (3)	0.0376	
C55	1.0136 (6)	0.27876 (19)	0.0977 (3)	0.0430	
C56	1.0058 (6)	0.28569 (19)	0.1671 (3)	0.0411	
C57	0.9162 (5)	0.26396 (17)	0.2072 (3)	0.0328	
O7	0.9078 (4)	0.27265 (13)	0.27508 (19)	0.0496	
O8	0.5679 (3)	0.16633 (11)	0.36129 (18)	0.0346	
C58	0.5740 (6)	0.1690 (2)	0.4332 (3)	0.0454	
P2	0.84347 (12)	0.14393 (4)	0.30527 (6)	0.0237	
C59	0.9067 (5)	0.11364 (16)	0.2334 (2)	0.0245	
C60	0.8667 (5)	0.12224 (17)	0.1664 (3)	0.0309	
C61	0.9140 (6)	0.09914 (18)	0.1123 (3)	0.0351	
C62	0.9999 (6)	0.06725 (19)	0.1250 (3)	0.0411	
C63	1.0381 (5)	0.05808 (18)	0.1910 (3)	0.0390	
C64	0.9927 (5)	0.08123 (17)	0.2453 (3)	0.0309	
C65	0.8028 (5)	0.10430 (16)	0.3673 (2)	0.0270	
C66	0.7012 (5)	0.07760 (18)	0.3496 (3)	0.0353	
C67	0.6680 (6)	0.04605 (18)	0.3925 (3)	0.0396	
C68	0.7342 (6)	0.0408 (2)	0.4543 (3)	0.0434	
C69	0.8325 (6)	0.0677 (2)	0.4733 (3)	0.0488	
C70	0.8689 (5)	0.0992 (2)	0.4296 (3)	0.0396	
C71	0.9885 (5)	0.16904 (16)	0.3422 (2)	0.0262	
C72	0.9667 (5)	0.19405 (19)	0.3990 (3)	0.0372	
C73	1.0719 (6)	0.2129 (2)	0.4330 (3)	0.0434	
C74	1.2007 (6)	0.20680 (19)	0.4107 (3)	0.0421	
C75	1.2234 (6)	0.18277 (18)	0.3542 (3)	0.0389	
C76	1.1182 (5)	0.16410 (16)	0.3200 (3)	0.0293	
H31	0.2729	0.4390	0.4232	0.0514*	
H71	0.8482	0.2609	0.2969	0.0742*	
H21	0.5361	0.4835	0.4581	0.0421*	
H2452	0.7422	0.4617	0.4979	0.0513*	
H41	0.8558	0.4108	0.4413	0.0530*	
H51	0.7582	0.3798	0.3462	0.0462*	
H2453	0.6262	0.3499	0.2742	0.0481*	
H91	0.6156	0.2996	0.1981	0.0502*	
H101	0.5316	0.2660	0.1036	0.0590*	
H111	0.3473	0.2922	0.0470	0.0569*	
H121	0.2513	0.3519	0.0807	0.0441*	
H151	0.0713	0.3344	0.3140	0.0389*	
H161	−0.0335	0.3020	0.4020	0.0459*	
H171	−0.0163	0.3258	0.5138	0.0437*	
H181	0.1145	0.3827	0.5369	0.0393*	
H201	0.5826	0.4920	0.1712	0.1122*	
H203	0.6414	0.4601	0.2221	0.1121*	
H202	0.5735	0.4457	0.1541	0.1123*	
H221	−0.0325	0.4045	0.2577	0.0491*	
H231	−0.1684	0.3625	0.1935	0.0549*	
H241	−0.1880	0.3727	0.0751	0.0649*	
H251	−0.0692	0.4241	0.0227	0.0711*	
H261	0.0650	0.4652	0.0869	0.0453*	
H281	0.3180	0.4826	0.1170	0.0376*	
H291	0.3679	0.5379	0.0470	0.0482*	
H301	0.2756	0.6000	0.0681	0.0522*	
H311	0.1293	0.6082	0.1579	0.0514*	
H321	0.0796	0.5539	0.2306	0.0399*	
H341	0.1833	0.4956	0.3710	0.0337*	
H351	0.0468	0.5264	0.4526	0.0330*	
H361	−0.1752	0.5396	0.4293	0.0436*	
H371	−0.2608	0.5223	0.3240	0.0449*	
H381	−0.1322	0.4902	0.2428	0.0404*	
H401	0.7335	0.2919	0.4368	0.0421*	
H411	0.5731	0.3383	0.4667	0.0648*	
H421	0.3643	0.3361	0.4165	0.0589*	
H431	0.3196	0.2898	0.3310	0.0620*	
H471	0.2176	0.2202	0.1557	0.0651*	
H481	0.1399	0.1688	0.0885	0.0800*	
H491	0.2579	0.1081	0.0835	0.0719*	
H501	0.4457	0.0993	0.1478	0.0450*	
H531	0.7926	0.2084	0.0845	0.0392*	
H541	0.9359	0.2465	0.0180	0.0459*	
H551	1.0748	0.2937	0.0719	0.0517*	
H561	1.0616	0.3051	0.1869	0.0487*	
H582	0.4868	0.1631	0.4514	0.0674*	
H581	0.6012	0.1960	0.4479	0.0674*	
H583	0.6377	0.1494	0.4513	0.0672*	
H601	0.8080	0.1438	0.1584	0.0379*	
H611	0.8878	0.1054	0.0679	0.0419*	
H621	1.0312	0.0518	0.0883	0.0500*	
H631	1.0949	0.0363	0.1987	0.0452*	
H641	1.0190	0.0747	0.2903	0.0345*	
H661	0.6552	0.0811	0.3079	0.0415*	
H671	0.6011	0.0278	0.3799	0.0469*	
H681	0.7119	0.0192	0.4826	0.0540*	
H691	0.8739	0.0645	0.5156	0.0578*	
H701	0.9377	0.1165	0.4424	0.0458*	
H721	0.8809	0.1986	0.4147	0.0439*	
H731	1.0535	0.2295	0.4703	0.0488*	
H741	1.2725	0.2186	0.4343	0.0527*	
H751	1.3101	0.1788	0.3386	0.0487*	
H761	1.1369	0.1476	0.2821	0.0347*	
N203	0.4631 (14)	0.2133 (4)	0.2461 (7)	0.0287	0.2708
C245	0.4222 (16)	0.2442 (5)	0.2775 (7)	0.0289	0.2708
H1451	0.3334	0.2472	0.2359	0.0398*	0.7292
H2451	0.3385	0.2555	0.2647	0.0398*	0.2708

### Atomic displacement parameters (Å^2^)

**Table:**

	*U*^11^	*U*^22^	*U*^33^	*U*^12^	*U*^13^	*U*^23^
Re1	0.02088 (10)	0.03125 (12)	0.02539 (11)	0.00382 (9)	0.00238 (8)	0.00394 (9)
O1	0.0259 (18)	0.030 (2)	0.037 (2)	−0.0006 (15)	−0.0077 (15)	0.0008 (16)
C1	0.020 (2)	0.030 (3)	0.027 (3)	−0.004 (2)	−0.0071 (19)	0.007 (2)
C2	0.037 (3)	0.038 (3)	0.031 (3)	−0.006 (2)	0.000 (2)	−0.004 (2)
C3	0.038 (3)	0.061 (4)	0.029 (3)	−0.015 (3)	−0.011 (2)	0.007 (3)
C4	0.029 (3)	0.051 (4)	0.054 (4)	−0.001 (2)	−0.007 (2)	0.016 (3)
C5	0.032 (3)	0.037 (3)	0.048 (3)	0.006 (2)	0.003 (2)	0.008 (2)
C6	0.028 (3)	0.032 (3)	0.029 (3)	−0.006 (2)	−0.002 (2)	0.004 (2)
N1	0.035 (2)	0.036 (3)	0.035 (2)	0.003 (2)	0.0063 (19)	0.005 (2)
C7	0.043 (3)	0.036 (3)	0.040 (3)	0.005 (2)	0.013 (2)	0.006 (2)
C8	0.036 (3)	0.034 (3)	0.027 (3)	−0.005 (2)	0.003 (2)	−0.001 (2)
C9	0.045 (3)	0.039 (3)	0.036 (3)	0.005 (2)	0.008 (2)	0.007 (2)
C10	0.060 (4)	0.042 (4)	0.036 (3)	0.013 (3)	0.012 (3)	−0.003 (2)
C11	0.061 (4)	0.052 (4)	0.030 (3)	−0.001 (3)	0.005 (3)	−0.012 (3)
C12	0.037 (3)	0.051 (4)	0.025 (3)	0.003 (2)	0.004 (2)	0.002 (2)
C13	0.041 (3)	0.031 (3)	0.028 (3)	0.001 (2)	0.013 (2)	0.001 (2)
O2	0.039 (2)	0.040 (2)	0.0260 (19)	0.0152 (17)	0.0013 (15)	−0.0028 (16)
N2	0.019 (2)	0.030 (2)	0.024 (2)	0.0037 (17)	−0.0035 (16)	−0.0042 (17)
C14	0.021 (2)	0.029 (3)	0.025 (2)	0.002 (2)	0.0053 (19)	0.000 (2)
C15	0.028 (3)	0.032 (3)	0.034 (3)	−0.001 (2)	0.005 (2)	−0.003 (2)
C16	0.029 (3)	0.037 (3)	0.047 (3)	−0.007 (2)	0.007 (2)	0.000 (2)
C17	0.035 (3)	0.035 (3)	0.039 (3)	0.002 (2)	0.009 (2)	0.006 (2)
C18	0.033 (3)	0.038 (3)	0.026 (3)	0.003 (2)	0.005 (2)	−0.003 (2)
C19	0.022 (2)	0.027 (3)	0.027 (3)	0.002 (2)	0.0003 (19)	0.002 (2)
O3	0.0334 (19)	0.036 (2)	0.0252 (18)	−0.0073 (16)	0.0014 (15)	−0.0004 (16)
O4	0.0212 (18)	0.047 (2)	0.049 (2)	0.0023 (16)	0.0080 (16)	0.0198 (18)
C20	0.036 (3)	0.093 (5)	0.098 (5)	0.011 (3)	0.013 (3)	0.042 (3)
P1	0.0205 (6)	0.0314 (8)	0.0185 (6)	0.0014 (5)	−0.0006 (5)	−0.0006 (6)
C21	0.020 (2)	0.033 (3)	0.026 (3)	0.004 (2)	−0.0038 (19)	−0.008 (2)
C22	0.028 (3)	0.049 (4)	0.040 (3)	0.000 (2)	−0.009 (2)	0.003 (2)
C23	0.039 (3)	0.042 (4)	0.058 (4)	−0.009 (2)	0.004 (3)	−0.004 (3)
C24	0.050 (4)	0.058 (4)	0.058 (4)	−0.015 (3)	−0.015 (3)	−0.021 (3)
C25	0.067 (4)	0.071 (5)	0.032 (3)	−0.013 (3)	−0.012 (3)	−0.013 (3)
C26	0.045 (3)	0.044 (3)	0.029 (3)	−0.007 (2)	−0.007 (2)	−0.003 (2)
C27	0.023 (2)	0.030 (3)	0.022 (2)	0.001 (2)	−0.0038 (19)	0.001 (2)
C28	0.028 (3)	0.044 (3)	0.022 (2)	−0.001 (2)	−0.006 (2)	0.000 (2)
C29	0.040 (3)	0.052 (4)	0.025 (3)	−0.013 (3)	−0.001 (2)	0.001 (2)
C30	0.050 (3)	0.037 (3)	0.038 (3)	−0.011 (3)	−0.008 (2)	0.011 (2)
C31	0.043 (3)	0.027 (3)	0.052 (3)	−0.001 (2)	−0.004 (3)	0.005 (2)
C32	0.031 (3)	0.034 (3)	0.034 (3)	0.000 (2)	0.001 (2)	−0.002 (2)
C33	0.024 (2)	0.027 (3)	0.021 (2)	0.0042 (19)	0.0030 (19)	0.0036 (19)
C34	0.028 (3)	0.032 (3)	0.022 (2)	0.001 (2)	−0.002 (2)	0.001 (2)
C35	0.037 (3)	0.030 (3)	0.021 (2)	−0.001 (2)	−0.002 (2)	−0.005 (2)
C36	0.040 (3)	0.036 (3)	0.030 (3)	0.004 (2)	0.011 (2)	−0.003 (2)
C37	0.027 (3)	0.046 (4)	0.044 (3)	0.007 (2)	0.009 (2)	−0.002 (2)
C38	0.029 (3)	0.047 (3)	0.027 (3)	0.004 (2)	−0.002 (2)	−0.002 (2)
Re2	0.02128 (11)	0.03115 (13)	0.03394 (12)	0.00348 (9)	0.00380 (8)	0.00742 (10)
O5	0.0309 (19)	0.036 (2)	0.040 (2)	0.0039 (16)	0.0085 (16)	0.0016 (17)
C39	0.026 (3)	0.029 (3)	0.026 (3)	−0.002 (2)	0.005 (2)	−0.002 (2)
C40	0.037 (3)	0.041 (3)	0.030 (3)	−0.013 (2)	0.001 (2)	−0.002 (2)
C41	0.085 (4)	0.029 (3)	0.036 (3)	−0.011 (3)	0.016 (3)	−0.010 (2)
C42	0.060 (4)	0.050 (4)	0.054 (4)	0.030 (3)	0.019 (3)	0.015 (3)
C43	0.041 (3)	0.065 (4)	0.043 (3)	0.014 (3)	0.001 (2)	0.021 (3)
C44	0.033 (2)	0.030 (2)	0.027 (2)	−0.0051 (19)	−0.0010 (18)	0.0018 (18)
N103	0.027 (2)	0.026 (3)	0.031 (2)	0.0012 (19)	0.0012 (19)	0.0043 (19)
C145	0.032 (3)	0.029 (3)	0.034 (3)	0.003 (2)	−0.002 (2)	0.001 (2)
C46	0.032 (2)	0.030 (2)	0.030 (2)	−0.0026 (19)	−0.0023 (18)	0.0005 (18)
C47	0.041 (3)	0.069 (4)	0.051 (4)	0.022 (3)	0.008 (3)	0.024 (3)
C48	0.036 (3)	0.127 (6)	0.038 (3)	−0.022 (3)	−0.020 (3)	0.010 (3)
C49	0.061 (4)	0.089 (5)	0.029 (3)	−0.041 (3)	0.003 (3)	−0.011 (3)
C50	0.046 (3)	0.040 (3)	0.031 (3)	−0.008 (2)	0.009 (2)	−0.008 (2)
C51	0.025 (3)	0.039 (3)	0.026 (3)	−0.005 (2)	0.000 (2)	0.004 (2)
O6	0.0297 (19)	0.030 (2)	0.043 (2)	−0.0045 (15)	−0.0053 (16)	0.0109 (16)
N4	0.025 (2)	0.032 (3)	0.030 (2)	0.0013 (18)	0.0009 (17)	0.0028 (18)
C52	0.027 (3)	0.024 (3)	0.030 (3)	0.005 (2)	−0.001 (2)	0.005 (2)
C53	0.035 (3)	0.032 (3)	0.034 (3)	−0.002 (2)	−0.004 (2)	0.001 (2)
C54	0.043 (3)	0.043 (3)	0.028 (3)	0.004 (2)	0.007 (2)	0.008 (2)
C55	0.044 (3)	0.047 (4)	0.038 (3)	−0.004 (3)	0.012 (2)	0.011 (2)
C56	0.037 (3)	0.040 (3)	0.047 (3)	−0.011 (2)	0.005 (2)	0.002 (2)
C57	0.036 (3)	0.031 (3)	0.031 (3)	−0.003 (2)	0.009 (2)	−0.002 (2)
O7	0.056 (3)	0.057 (3)	0.036 (2)	−0.027 (2)	0.0111 (18)	−0.0117 (19)
O8	0.0298 (19)	0.037 (2)	0.037 (2)	0.0049 (16)	0.0123 (15)	0.0092 (16)
C58	0.043 (3)	0.058 (4)	0.036 (3)	0.012 (3)	0.012 (2)	0.005 (3)
P2	0.0213 (6)	0.0301 (8)	0.0197 (6)	0.0015 (5)	−0.0002 (5)	−0.0002 (5)
C59	0.022 (2)	0.029 (3)	0.023 (2)	0.000 (2)	0.0010 (19)	−0.003 (2)
C60	0.030 (3)	0.036 (3)	0.026 (3)	−0.005 (2)	−0.001 (2)	−0.001 (2)
C61	0.042 (3)	0.046 (3)	0.018 (2)	−0.009 (2)	0.002 (2)	−0.005 (2)
C62	0.038 (3)	0.047 (4)	0.038 (3)	−0.014 (2)	0.012 (2)	−0.019 (2)
C63	0.029 (3)	0.036 (3)	0.052 (3)	0.005 (2)	0.002 (2)	−0.012 (2)
C64	0.027 (3)	0.037 (3)	0.029 (3)	0.003 (2)	−0.003 (2)	−0.001 (2)
C65	0.024 (2)	0.033 (3)	0.024 (2)	0.002 (2)	−0.0009 (19)	0.000 (2)
C66	0.035 (3)	0.041 (3)	0.030 (3)	0.001 (2)	−0.003 (2)	0.007 (2)
C67	0.045 (3)	0.038 (3)	0.036 (3)	−0.009 (2)	0.001 (2)	0.005 (2)
C68	0.050 (3)	0.047 (4)	0.034 (3)	0.002 (3)	0.002 (2)	0.016 (2)
C69	0.038 (3)	0.076 (4)	0.032 (3)	−0.005 (3)	−0.002 (2)	0.017 (3)
C70	0.030 (3)	0.055 (4)	0.033 (3)	−0.002 (2)	−0.006 (2)	0.009 (2)
C71	0.030 (3)	0.026 (3)	0.022 (2)	−0.001 (2)	−0.0020 (19)	0.002 (2)
C72	0.029 (3)	0.054 (4)	0.028 (3)	−0.002 (2)	−0.002 (2)	−0.010 (2)
C73	0.044 (3)	0.054 (4)	0.032 (3)	−0.002 (3)	−0.005 (2)	−0.016 (2)
C74	0.039 (3)	0.045 (4)	0.042 (3)	−0.012 (2)	−0.007 (2)	0.001 (3)
C75	0.030 (3)	0.039 (3)	0.048 (3)	−0.008 (2)	0.006 (2)	0.004 (2)
C76	0.027 (3)	0.030 (3)	0.031 (3)	−0.004 (2)	0.006 (2)	0.001 (2)
N203	0.029 (3)	0.028 (4)	0.029 (3)	0.001 (3)	−0.001 (3)	0.002 (3)
C245	0.031 (4)	0.029 (4)	0.027 (3)	−0.002 (3)	−0.002 (3)	0.001 (3)

### Geometric parameters (Å, °)

**Table:**

Re1—O1	2.100 (3)	Re2—O5	2.084 (4)
Re1—N1	2.122 (5)	Re2—N103	2.107 (6)
Re1—O2	2.027 (3)	Re2—O6	2.021 (4)
Re1—N2	1.760 (4)	Re2—N4	1.754 (4)
Re1—O4	1.921 (3)	Re2—O8	1.936 (3)
Re1—P1	2.4220 (13)	Re2—P2	2.4272 (13)
O1—C1	1.354 (6)	O5—C39	1.347 (6)
C1—C2	1.396 (7)	C39—C40	1.390 (7)
C1—C6	1.401 (7)	C39—C44	1.405 (7)
C2—C3	1.382 (8)	C40—C41	1.384 (8)
C2—H21	0.939	C40—H401	0.943
C3—C4	1.372 (9)	C41—C42	1.375 (9)
C3—H2452	0.947	C41—H411	0.929
C4—C5	1.386 (8)	C42—C43	1.377 (9)
C4—H41	0.931	C42—H421	0.919
C5—C6	1.386 (7)	C43—C44	1.376 (8)
C5—H51	0.922	C43—H431	0.926
C6—N1	1.444 (7)	C44—C245	1.443 (9)
N1—C7	1.254 (7)	C44—N103	1.478 (6)
C7—C8	1.461 (8)	N103—C145	1.275 (7)
C7—H2453	0.945	C145—C46	1.434 (7)
C8—C9	1.408 (8)	C145—H1451	0.950
C8—C13	1.420 (8)	C46—C47	1.421 (8)
C9—C10	1.365 (8)	C46—C51	1.416 (7)
C9—H91	0.941	C46—N203	1.469 (9)
C10—C11	1.389 (9)	C46—C47	1.421 (8)
C10—H101	0.924	C46—C51	1.416 (7)
C11—C12	1.373 (8)	C47—C48	1.372 (10)
C11—H111	0.929	C47—H471	0.927
C12—C13	1.400 (8)	C48—C49	1.366 (11)
C12—H121	0.938	C48—H481	0.940
C13—O2	1.326 (6)	C49—C50	1.348 (9)
N2—C14	1.381 (6)	C49—H491	0.937
C14—C15	1.403 (7)	C50—C51	1.390 (8)
C14—C19	1.398 (7)	C50—H501	0.934
C15—C16	1.374 (7)	C51—O6	1.334 (6)
C15—H151	0.939	N4—C52	1.365 (6)
C16—C17	1.388 (8)	C52—C53	1.413 (7)
C16—H161	0.923	C52—C57	1.401 (7)
C17—C18	1.381 (8)	C53—C54	1.377 (7)
C17—H171	0.938	C53—H531	0.931
C18—C19	1.396 (7)	C54—C55	1.377 (8)
C18—H181	0.927	C54—H541	0.947
C19—O3	1.347 (6)	C55—C56	1.372 (8)
O3—H31	0.814	C55—H551	0.934
O4—C20	1.327 (7)	C56—C57	1.390 (7)
C20—H201	0.961	C56—H561	0.932
C20—H203	0.957	C57—O7	1.354 (6)
C20—H202	0.959	O7—H71	0.830
P1—C21	1.848 (5)	O8—C58	1.404 (6)
P1—C27	1.828 (5)	C58—H582	0.965
P1—C33	1.822 (5)	C58—H581	0.973
C21—C22	1.371 (8)	C58—H583	0.973
C21—C26	1.376 (7)	P2—C59	1.833 (5)
C22—C23	1.377 (8)	P2—C65	1.826 (5)
C22—H221	0.941	P2—C71	1.819 (5)
C23—C24	1.386 (9)	C59—C60	1.393 (7)
C23—H231	0.935	C59—C64	1.391 (7)
C24—C25	1.369 (9)	C60—C61	1.384 (7)
C24—H241	0.934	C60—H601	0.936
C25—C26	1.378 (8)	C61—C62	1.380 (8)
C25—H251	0.942	C61—H611	0.926
C26—H261	0.926	C62—C63	1.373 (8)
C27—C28	1.398 (7)	C62—H621	0.933
C27—C32	1.382 (7)	C63—C64	1.382 (7)
C28—C29	1.377 (8)	C63—H631	0.928
C28—H281	0.940	C64—H641	0.938
C29—C30	1.384 (8)	C65—C66	1.389 (7)
C29—H291	0.926	C65—C70	1.390 (7)
C30—C31	1.368 (8)	C66—C67	1.375 (7)
C30—H301	0.932	C66—H661	0.940
C31—C32	1.392 (8)	C67—C68	1.382 (8)
C31—H311	0.928	C67—H671	0.932
C32—H321	0.937	C68—C69	1.375 (9)
C33—C34	1.387 (7)	C68—H681	0.930
C33—C38	1.397 (7)	C69—C70	1.392 (8)
C34—C35	1.391 (7)	C69—H691	0.927
C34—H341	0.938	C70—H701	0.929
C35—C36	1.386 (7)	C71—C72	1.396 (7)
C35—H351	0.929	C71—C76	1.381 (7)
C36—C37	1.372 (8)	C72—C73	1.389 (8)
C36—H361	0.934	C72—H721	0.925
C37—C38	1.384 (7)	C73—C74	1.377 (8)
C37—H371	0.912	C73—H731	0.929
C38—H381	0.934	C74—C75	1.375 (8)
Re2—O5	2.084 (4)	C74—H741	0.938
Re2—O6	2.021 (4)	C75—C76	1.388 (7)
Re2—N4	1.754 (4)	C75—H751	0.931
Re2—O8	1.936 (3)	C76—H761	0.936
Re2—P2	2.4272 (13)	N203—C245	1.256 (9)
Re2—N203	2.214 (14)	C245—H2451	0.950
			
O1—Re1—N1	79.17 (16)	P2—Re2—N203	162.8 (3)
O1—Re1—O2	168.92 (14)	O5—Re2—N103	76.85 (17)
N1—Re1—O2	92.33 (16)	O5—Re2—O6	168.12 (14)
O1—Re1—N2	89.27 (16)	N103—Re2—O6	94.75 (18)
N1—Re1—N2	97.22 (17)	O5—Re2—N4	89.90 (17)
O2—Re1—N2	98.93 (17)	N103—Re2—N4	97.1 (2)
O1—Re1—O4	83.63 (16)	O6—Re2—N4	99.58 (17)
N1—Re1—O4	83.85 (16)	O5—Re2—O8	84.34 (15)
O2—Re1—O4	88.43 (16)	N103—Re2—O8	83.63 (19)
N2—Re1—O4	172.49 (18)	O6—Re2—O8	86.41 (15)
O1—Re1—P1	101.51 (10)	N4—Re2—O8	173.86 (18)
N1—Re1—P1	172.60 (12)	O5—Re2—P2	95.64 (10)
O2—Re1—P1	86.01 (10)	N103—Re2—P2	169.50 (15)
N2—Re1—P1	90.16 (13)	O6—Re2—P2	91.53 (11)
O4—Re1—P1	88.90 (11)	N4—Re2—P2	90.08 (14)
Re1—O1—C1	112.1 (3)	O8—Re2—P2	88.38 (11)
O1—C1—C2	119.2 (5)	Re2—O5—C39	115.3 (3)
O1—C1—C6	122.6 (4)	O5—C39—C40	119.6 (5)
C2—C1—C6	118.2 (5)	O5—C39—C44	122.1 (5)
C1—C2—C3	120.2 (5)	C40—C39—C44	118.3 (5)
C1—C2—H21	119.4	C39—C40—C41	120.7 (5)
C3—C2—H21	120.4	C39—C40—H401	119.8
C2—C3—C4	121.4 (5)	C41—C40—H401	119.5
C2—C3—H2452	119.1	C40—C41—C42	120.1 (6)
C4—C3—H2452	119.5	C40—C41—H411	118.8
C3—C4—C5	119.2 (5)	C42—C41—H411	121.1
C3—C4—H41	120.4	C41—C42—C43	119.9 (6)
C5—C4—H41	120.3	C41—C42—H421	119.5
C4—C5—C6	120.2 (6)	C43—C42—H421	120.6
C4—C5—H51	120.7	C42—C43—C44	120.7 (6)
C6—C5—H51	119.0	C42—C43—H431	119.5
C1—C6—C5	120.7 (5)	C44—C43—H431	119.8
C1—C6—N1	112.5 (4)	C39—C44—C43	120.1 (5)
C5—C6—N1	126.9 (5)	C39—C44—C245	143.1 (8)
Re1—N1—C6	113.3 (3)	C43—C44—C245	96.7 (8)
Re1—N1—C7	123.3 (4)	C39—C44—C43	120.1 (5)
C6—N1—C7	123.4 (5)	C39—C44—N103	108.6 (5)
N1—C7—C8	126.0 (6)	C43—C44—N103	131.2 (5)
N1—C7—H2453	117.7	C44—N103—Re2	116.4 (4)
C8—C7—H2453	116.3	C44—N103—C145	121.1 (5)
C7—C8—C9	113.9 (5)	Re2—N103—C145	122.5 (5)
C7—C8—C13	126.6 (5)	N103—C145—C46	122.1 (6)
C9—C8—C13	119.5 (5)	N103—C145—H1451	119.4
C8—C9—C10	121.9 (6)	C46—C145—H1451	118.4
C8—C9—H91	118.8	C47—C46—C51	118.2 (5)
C10—C9—H91	119.2	C47—C46—N203	140.8 (8)
C9—C10—C11	118.3 (6)	C51—C46—N203	101.0 (7)
C9—C10—H101	121.1	C145—C46—C47	109.3 (5)
C11—C10—H101	120.6	C145—C46—C51	132.2 (5)
C10—C11—C12	121.5 (6)	C47—C46—C51	118.2 (5)
C10—C11—H111	119.3	C46—C47—C48	121.0 (6)
C12—C11—H111	119.2	C46—C47—H471	120.3
C11—C12—C13	121.5 (6)	C48—C47—H471	118.7
C11—C12—H121	119.4	C47—C48—C49	119.5 (6)
C13—C12—H121	119.0	C47—C48—H481	119.1
C8—C13—C12	117.2 (5)	C49—C48—H481	121.3
C8—C13—O2	126.1 (5)	C48—C49—C50	121.0 (6)
C12—C13—O2	116.7 (5)	C48—C49—H491	120.3
Re1—O2—C13	123.0 (3)	C50—C49—H491	118.7
Re1—N2—C14	167.1 (4)	C49—C50—C51	122.5 (6)
N2—C14—C15	120.6 (4)	C49—C50—H501	120.7
N2—C14—C19	120.0 (4)	C51—C50—H501	116.8
C15—C14—C19	119.3 (5)	C46—C51—C50	117.9 (5)
C14—C15—C16	120.7 (5)	C46—C51—O6	124.3 (5)
C14—C15—H151	120.6	C50—C51—O6	117.8 (5)
C16—C15—H151	118.7	Re2—O6—C51	121.1 (3)
C15—C16—C17	119.7 (5)	Re2—N4—C52	167.3 (4)
C15—C16—H161	119.3	N4—C52—C53	121.7 (5)
C17—C16—H161	121.1	N4—C52—C57	120.2 (5)
C16—C17—C18	120.7 (5)	C53—C52—C57	118.1 (5)
C16—C17—H171	118.9	C52—C53—C54	121.2 (5)
C18—C17—H171	120.5	C52—C53—H531	119.0
C17—C18—C19	120.1 (5)	C54—C53—H531	119.7
C17—C18—H181	120.8	C53—C54—C55	119.3 (5)
C19—C18—H181	119.1	C53—C54—H541	119.7
C14—C19—C18	119.5 (5)	C55—C54—H541	121.0
C14—C19—O3	122.7 (4)	C54—C55—C56	121.1 (5)
C18—C19—O3	117.8 (4)	C54—C55—H551	120.3
C19—O3—H31	115.0	C56—C55—H551	118.6
Re1—O4—C20	141.5 (4)	C55—C56—C57	120.4 (6)
O4—C20—H201	108.9	C55—C56—H561	119.0
O4—C20—H203	111.0	C57—C56—H561	120.6
H201—C20—H203	108.9	C52—C57—C56	119.9 (5)
O4—C20—H202	110.0	C52—C57—O7	121.4 (5)
H201—C20—H202	109.0	C56—C57—O7	118.7 (5)
H203—C20—H202	109.0	C57—O7—H71	116.5
Re1—P1—C21	110.18 (17)	Re2—O8—C58	138.8 (4)
Re1—P1—C27	115.27 (16)	O8—C58—H582	108.4
C21—P1—C27	104.9 (2)	O8—C58—H581	111.3
Re1—P1—C33	118.60 (16)	H582—C58—H581	109.4
C21—P1—C33	102.8 (2)	O8—C58—H583	110.2
C27—P1—C33	103.6 (2)	H582—C58—H583	109.0
P1—C21—C22	117.9 (4)	H581—C58—H583	108.5
P1—C21—C26	122.7 (4)	Re2—P2—C59	117.23 (16)
C22—C21—C26	119.4 (5)	Re2—P2—C65	112.86 (16)
C21—C22—C23	120.6 (5)	C59—P2—C65	101.1 (2)
C21—C22—H221	119.9	Re2—P2—C71	114.13 (17)
C23—C22—H221	119.5	C59—P2—C71	105.8 (2)
C22—C23—C24	119.5 (6)	C65—P2—C71	104.1 (2)
C22—C23—H231	119.8	P2—C59—C60	120.3 (4)
C24—C23—H231	120.6	P2—C59—C64	120.4 (4)
C23—C24—C25	120.0 (6)	C60—C59—C64	119.3 (5)
C23—C24—H241	120.5	C59—C60—C61	120.1 (5)
C25—C24—H241	119.5	C59—C60—H601	119.3
C24—C25—C26	119.9 (6)	C61—C60—H601	120.6
C24—C25—H251	120.7	C60—C61—C62	119.9 (5)
C26—C25—H251	119.4	C60—C61—H611	119.4
C25—C26—C21	120.5 (6)	C62—C61—H611	120.8
C25—C26—H261	118.4	C61—C62—C63	120.5 (5)
C21—C26—H261	121.1	C61—C62—H621	119.3
P1—C27—C28	119.5 (4)	C63—C62—H621	120.2
P1—C27—C32	121.5 (4)	C62—C63—C64	120.1 (5)
C28—C27—C32	119.0 (5)	C62—C63—H631	119.4
C27—C28—C29	120.5 (5)	C64—C63—H631	120.5
C27—C28—H281	119.0	C59—C64—C63	120.1 (5)
C29—C28—H281	120.5	C59—C64—H641	120.3
C28—C29—C30	120.1 (5)	C63—C64—H641	119.6
C28—C29—H291	121.0	P2—C65—C66	117.1 (4)
C30—C29—H291	118.9	P2—C65—C70	123.8 (4)
C29—C30—C31	119.9 (5)	C66—C65—C70	119.1 (5)
C29—C30—H301	119.0	C65—C66—C67	120.5 (5)
C31—C30—H301	121.1	C65—C66—H661	119.6
C30—C31—C32	120.6 (6)	C67—C66—H661	119.9
C30—C31—H311	119.0	C66—C67—C68	120.4 (6)
C32—C31—H311	120.3	C66—C67—H671	120.2
C31—C32—C27	119.9 (5)	C68—C67—H671	119.4
C31—C32—H321	120.6	C67—C68—C69	119.7 (6)
C27—C32—H321	119.5	C67—C68—H681	119.7
P1—C33—C34	119.4 (4)	C69—C68—H681	120.6
P1—C33—C38	121.4 (4)	C68—C69—C70	120.4 (5)
C34—C33—C38	119.0 (5)	C68—C69—H691	118.9
C33—C34—C35	119.6 (5)	C70—C69—H691	120.7
C33—C34—H341	120.0	C69—C70—C65	119.8 (5)
C35—C34—H341	120.4	C69—C70—H701	119.4
C34—C35—C36	121.0 (5)	C65—C70—H701	120.8
C34—C35—H351	120.0	P2—C71—C72	117.1 (4)
C36—C35—H351	118.9	P2—C71—C76	125.1 (4)
C35—C36—C37	119.3 (5)	C72—C71—C76	117.8 (5)
C35—C36—H361	120.7	C71—C72—C73	121.4 (5)
C37—C36—H361	120.0	C71—C72—H721	120.3
C36—C37—C38	120.4 (5)	C73—C72—H721	118.3
C36—C37—H371	119.7	C72—C73—C74	119.7 (5)
C38—C37—H371	119.9	C72—C73—H731	119.0
C33—C38—C37	120.7 (5)	C74—C73—H731	121.3
C33—C38—H381	119.7	C73—C74—C75	119.5 (5)
C37—C38—H381	119.6	C73—C74—H741	120.2
O5—Re2—O6	168.12 (14)	C75—C74—H741	120.2
O5—Re2—N4	89.90 (17)	C74—C75—C76	120.8 (5)
O6—Re2—N4	99.58 (17)	C74—C75—H751	120.0
O5—Re2—O8	84.34 (15)	C76—C75—H751	119.3
O6—Re2—O8	86.41 (15)	C75—C76—C71	120.8 (5)
N4—Re2—O8	173.86 (18)	C75—C76—H761	118.8
O5—Re2—P2	95.64 (10)	C71—C76—H761	120.4
O6—Re2—P2	91.53 (11)	Re2—N203—C46	121.1 (8)
N4—Re2—P2	90.08 (14)	Re2—N203—C245	115.4 (9)
O8—Re2—P2	88.38 (11)	C46—N203—C245	123.0 (11)
O5—Re2—N203	101.1 (3)	C44—C245—N203	121.8 (12)
O6—Re2—N203	71.3 (3)	C44—C245—H2451	119.5
N4—Re2—N203	94.1 (4)	N203—C245—H2451	118.7
O8—Re2—N203	89.1 (4)		

### Hydrogen-bond geometry (Å, °)

**Table:**

*D*—H···*A*	*D*—H	H···*A*	*D*···*A*	*D*—H···*A*
O3—H31···O1	0.81	1.93	2.693 (8)	156
O7—H71···O5	0.83	1.89	2.720 (8)	177
C34—H341···O1	0.94	2.53	3.397 (8)	153
C60—H601···N4	0.94	2.59	3.342 (8)	137
C72—H721···O5	0.92	2.38	2.983 (8)	123
